# The Decrease of Peripheral Blood CD4+ T Cells Indicates Abdominal Compartment Syndrome in Severe Acute Pancreatitis

**DOI:** 10.1371/journal.pone.0135768

**Published:** 2015-08-19

**Authors:** Yao Liu, Ling Wang, Zhifang Cai, Peng Zhao, Cijun Peng, Lijin Zhao, Chidan Wan

**Affiliations:** 1 Department of Hepatobiliary Surgery, Union Hospital, Tongji Medical College, Huazhong University of Science and Technology, Wuhan, Hubei Province, People’s Republic of China; 2 Department of Hepatobiliary and Pancreatic Surgery, Affiliated Hospital of Zunyi Medical College, Zunyi, Guizhou Province, People’s Republic of China; RWTH Aachen, GERMANY

## Abstract

**Objective:**

Few data are available on the role of T lymphocytes and inflammatory cytokines in abdominal compartment syndrome (ACS) in severe acute pancreatitis (SAP). We conducted a retrospective study to assess the risk factors associated with ACS in SAP.

**Methods:**

A total of 76 SAP patients who were admitted within 24 hours after symptom onset in our study. There were 36 patients suffering from ACS and 40 from intra-abdominal hypertension (IAH). On the 1^st^, 3^rd^ and 7^th^ days after hospital admission, the following variables were assessed: serum value of C-reactive protein (CRP), and the proportions of peripheral CD4^+^ and CD8^+^ T lymphocytes. Acute Physiology and Chronic Health Evaluation II (APACHE II) score, and computed tomography severity index (CTSI) score were assessed on days 1 and 7 after hospitalization.

**Results:**

Compared with the patients with IAH, ACS patients showed statistically higher CRP value on 7^th^ day after hospital admission, proportions of CD4^+^ T cells on days 1, 3, 7 and CD4^+^ / CD8^+^ ratio on day 1 were significantly lower (*P* < 0.05, respectively). A CD4^+^ T cell proportion of 30.3% on the 1^st^ day indicated ACS with an area under the curve (AUC) of 0.774, a sensitivity with 82.5% and specificity with 72.0%, respectively. Sensitivity / specificity for predicting ACS in SAP patients on day 1 was 70.0% / 68.0% for CD4^+^ / CD8^+^ ratio, 72.2% / 65.0% for APACHE II score.

**Conclusions:**

The reduction of peripheral blood CD4^+^ T lymphocytes is associated with ACS in SAP, and may act as a potential predictor of ACS in SAP.

## Introduction

Acute pancreatitis (AP) is a mild and self-limiting disease, and approximately 80% of AP patients recover without complications [1]. However, SAP accounts for around 20% of AP patients, and is associated with a mortality rate ranging from 36% to 50% [2–3]. Severe acute pancreatitis (SAP) is most commonly characterized by cytokine activation, pancreatic necrosis, systemic inflammatory response syndrome (SIRS), and multiple organ dysfunction syndrome (MODS) [4–5]. Prediction of AP severity and outcome is essential for timely treatment and prevention of complications, and remains need to be systematic studied. Radiological imaging procedures, multiple clinico-biochemical scores, and several biochemical markers, have been used to assess severity and outcome of AP [6–9].

Intra-abdominal hypertension (IAH) is defined as sustained increase of intra-abdominal pressure (IAP) > 12 mmHg, and abdominal compartment syndrome (ACS), a lethal complication of SAP, is defined as the combination of IAP > 20 mmHg and new-onset organ failure (OF) or acute worsening of existing OF [10]. De Waele *et al*. [11] found that IAP above 25 mmHg was detected in 30% of SAP patients, while IAP > 15 mmHg was found in 78% of SAP patients. In SAP patients, ACS has drawn more attention, because it has a mortality rate of 30–60% [3], and the diagnosis of ACS is difficult. The symptoms of ACS may resemble those of other complications, such as infected pancreatic necrosis, SIRS, and MODS [12]. The pathophysiology of ACS is considered to be directly associated with the pancreas inflammation, which initiates a cascade of acute peripancreatic fluid collections (APFC), capillary leakage syndrome (CLS), and paralytic ileus leading to an elevated IAP [11,13]. SAP is a critical risk factor for ACS, therefore, it is necessary to routinely monitor IAP in SAP patients according to the 2013 WSACS guidelines [10].

At present, it is suggested that the cytokine cascade from the innate immune system and the activated adaptive immune system (including CD4^+^ and CD8^+^ T lymphocytes) are essential to the development of SIRS in AP [4,14]. T lymphocytes are critical in the regulation of the adaptive immune system, and have a particular effect on innate immune system. In a mice model of AP, predominantly CD4^+^ T cells invaded the pancreas and infiltrated border acini [15]. Alterations of the immune systems in AP patients with IAH or even ACS should be thoroughly explored indicaed that ACS is related to higher mortality and morbidity rates compared to patients without ACS [11,13]. However, the detailed mechanism of ACS in SAP patients is still unclear. In this retrospective study, we intended to identify the role of T lymphocyte in the progression of ACS in SAP patients.

## Materials and Methods

### Patients

This retrospective study involved a total of 76 patients with SAP who were admitted to our institution from December 2012 to July 2014 within 24 hours after symptom onset.

The diagnostic criteria of SAP were based on the revised Atlanta classification [16]. Patients who develop persistent organ failure (POF) more than 48 hours and present one or more of the following features were included in the study: (1) an Acute Physiology and Chronic Health Evaluation II (APACHE II) score ≥ 8; (2) local complications, such as infected pancreatic necrosis, pancreatic abscess or pseudocyst. OF was defined as score ≥ 2 according to a modified Marshall scoring system (S1 Table) for one of the particular organ systems, such as respiratory, cardiovascular and renal [17].

The exclusion criteria included any of the followings: age < 18 years or > 80 years, pancreatitis induced by trauma or pregnant, a diagnosis of chronic or recurrent pancreatitis, a history of chronic pulmonary, renal or cardiovascular disease, and any surgical intervention taken in the first 3 days after hospital admission. The research was implemented based on the principles of the Declaration of Helsinki. The need for informed consent was waived by the Medical Ethics Committee of Affiliated Hospital of Zunyi Medical College, because the patients′ identification information in this study had been removed. The Institutional Ethics Review Board of Affiliated Hospital of Zunyi Medical College approved this study.

There were 36 patients suffering from ACS and 40 from IAH, respectively. SAP patients with 12 mmHg < IAP ≤ 20 mmHg were selected into the group of IAH, and patients with IAP > 20 mmHg were chosen into the group of ACS. IAP measurement was performed daily using a Foley catheter, which was inserted into the urinary bladder and instilled with 25 mL sterile saline (1 mm Hg = 1.36 cm H_2_O) with mid axillary line as level 0 [18]. The APACHE II scores [8] were recorded on days 1 and 7.

### Treatment

All patients received standard treatment according to the United Kingdom and Chinese Medical Association guidelines for the management of acute pancreatitis [19–20]. Fluid resuscitation followed international guidelines. Both colloids and crystalloids were used based on the assessment of patients. Parenteral nutrition was given, and once the gastrointestinal function recovered, enteral nutrition was necessary to be administrated as early as possible. Antibiotic prophylaxis was given for no more than 10 days, unless there were clinical symptoms of persistent sepsis. Noninvasive or invasive mechanical ventilation and dialysis were implemented when needed. The patient would experience an endoscopic retrograde cholangiopancreatography (ERCP), if confirmed of an obstruction in the ampulla of Vater or in the common bile duct by computed tomography (CT) scan. A contrast-enhanced CT scan was performed within 24 hours of admission before any surgical intervention, and repeated on day 7 to observe the morphology of SAP. A radiologist, blinded to the study, reviewed all CT scans. The CT findings on days 1 and 7 were graded using the CT severity index (CTSI) [6,21]. The infection of pancreatic necrosis was presumed when extraluminal gas emerged in the peripancreatic and / or pancreatic tissues on contrast-enhanced CT or when percutaneous fine-needle aspiration was positive for bacteria and / or fungi on Gram stain and culture [16].

### Sample collection and measurements

#### Serum CRP level

Serum CRP value was obtained on 1^st^, 3^rd^ and 7^th^ day after hospitalization, and measured by the Cobas Integra 800 (Roche, Basel, Switzerland) in the laboratory.

#### CD4+ and CD8+ T lymphocytes

Surface monocyte antigens of heparinized peripheral blood lymphocytes were determined using a flow cytometer (FACSCanto II, BD Bioscience, CA, USA) and double-stained (fluorescein isothiocyanate / phycoerythrin) monoclonal antibodies (BD Bioscience, CA, USA).

### Statistical Analysis

All statistical analysis was undertaken with SPSS 17.0 software (SPSS Inc, Chicago, IL). Data are shown as mean ± standard deviation (SD). Mann-Whitney U test, and Student’s t test were respectively used for non-normally and normally distributed variables in group comparisons. A chi-square test was used to compare categorical variables. Data between days 1, 3 and 7 were compared using Friedman repeated-measures ANOVA on ranks. *P* < 0.05 was considered statistically significant for all comparisons. Receiver-operating curves (ROC) were generated to calculate the cut-off values for optimal sensitivity and specificity.

## Results

### Baseline Characteristics

There were no differences in demographic data, listed in Table 1, between ACS (n = 36) and IAH (n = 40). Among the 36 ACS patients, persistent solitary organ failure (OF) appeared in 27 (75.0%) and persistent multiple OF in 9 (25.0%). Twenty-one patients in the group of ACS suffered from acute peripancreatic fluid collection (APFC), and 15 developed acute necrotic collection (ANC). Six patients with ACS developed pancreatic pseudocyst, and 5 developed walled-off necrosis (WON). In the 40 IAH patients, 34 (85.0%) suffered from persistent solitary OF and 6 (15.0%) with persistent multiple OF. Twenty-five IAH patients suffered from APFC, and 15 from ANC. Five patients developed pancreatic pseudocyst, and 6 developed WON. Infected pancreatic necrosis was found in 12 ACS patients and in 9 IAH ones, which was confirmed by positive fine-needle aspiration of necrotic tissue. Infection was observed from 5 to 19 days after hospitalization, with a median time of 11 days. Six patients with infected pancreatic necrosis were conservatively treated with percutaneous catheter drainage (PCD) of organized pancreatic necrosis, and no laparotomy was performed. The other 15 patients ultimately underwent pancreatic debridement and double drainage for the following reasons, with a mean operation time of 21 days: poor outcome of PCD, clinical deterioration that required immediate clear of infected pancreatic necrosis to improve sepsis, and simultaneous implementation of cholecystectomy or choledocholithotomy with open necrosectomy. All patients who only had single organ failure during hospitalization survived. Fifteen patients (9 ACS patients and 6 IAH ones) died due to persistent organ failure or infected necrosis during hospitalization, with a total mortality rate of 19.7%. There was no difference in mortality between the two groups (*P* = 0.274, Table 1).

**Table 1:** Demographic data and clinical characteristics of the SAP patients.

	ACS (n = 36)	IAH (n = 40)	*P*-value
Gender (male/female)	20 / 16	21 / 19	0.790
Age (years)	49.31 ± 8.33	53.3 ± 9.12	0.051
Etiology, n (%)			0.838
Biliary	15 (41.67%)	16 (40.0%)	
Alcoholic	7 (19.44%)	9 (22.5%)	
Hyperlipidemia	7 (19.44%)	9 (22.5%)	
Post ERCP pancreatitis	3 (8.33%)	1 (2.5%)	
Idiopathic	4 (11.11%)	5 (12.5%)	
Persistent organ failure (POF)			0.274
Solitary POF, n (%)	27 (75.0%)	34 (85.0%)	
Multiple POF, n (%)	9 (25.0%)	6 (15.0%)	
IAP (mmHg)	23.75 ± 2.72	16.68 ± 1.56	0.000
APACHE II score on day 1	10.50 ± 1.48	9.30 ± 1.56	0.001
APACHE II score on day 7	9.03 ± 3.01	7.15 ± 1.53	0.001
CTSI score on day 1	5.11 ± 0.92	4.90 ± 0.74	0.273
CTSI score on day 7	4.78 ± 1.71	3.73 ± 0.75	0.001
CRP on day 1	263.7 ± 74.2	253.5 ± 33.2	0.525
CRP on day 3	176.0 ± 60.9	172.5 ± 40.9	0.950
CRP on day 7	109.5 ± 49.2	88.8 ± 18.4	0.014
acute peripancreatic fluid collection (APFC)	21	25	0.711
acute necrotic collection (ANC)	15	15	0.711
Infected pancreatic necrosis	12	9	0.292
Surgical intervention	8	7	0.606
Mortality	9	6	0.274

Data are presented in either means and standard deviations or frequencies and percentages.

### Serum CRP level

**Fig 1:**
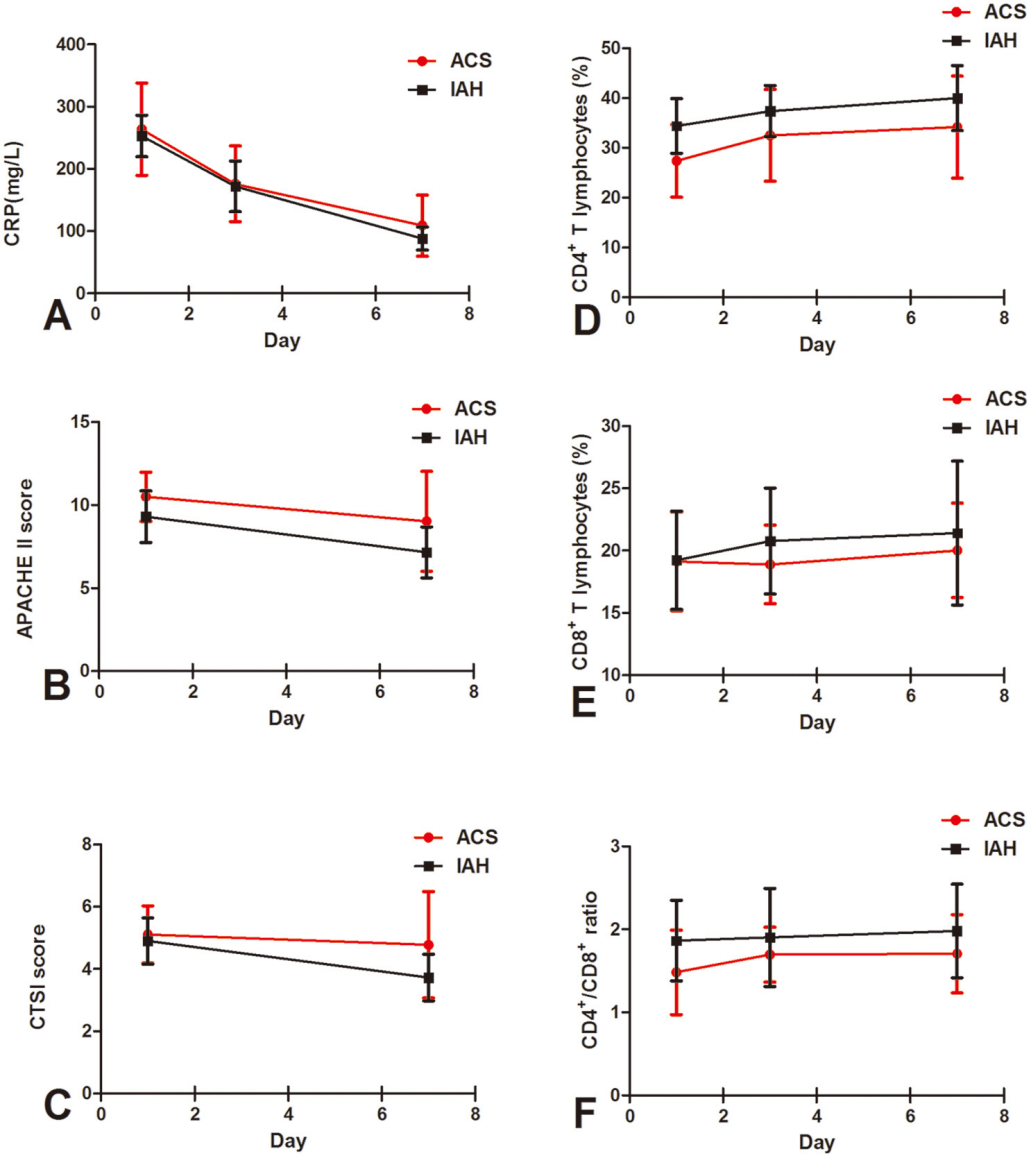
Sequential changes in the CRP value (A), APACHE II score (B), CTSI score (C), the proportion of CD4^+^ T lymphocytes (D), the proportion of CD8^+^ T lymphocytes (E) and CD4^+^ / CD8^+^ ratio (F) in the ACS and IAH groups. Data of CD4^+^ and CD8^+^ T cells were available in 25, 24 and 25 ACS patients on days 1, 3 and 7, respectively.

Serum peak value of CRP was noticed on day 1, and then rapidly decreased on days 3 and 7 in both ACS patients (263.7 ± 74.2 mg/L vs. 176.0 ± 60.9 mg/L and 109.5 ± 49.2 mg/L, *P* < 0.01) and IAH ones (253.5 ± 33.2 mg/L vs. 172.5 ± 40.9 mg/L and 88.8 ± 18.4 mg/L, *P* < 0.01). Serum CRP value on day 7 was statistically higher in the ACS group compared with the IAH group (*P* < 0.05, Table 1; Fig 1).

### CD4^+^ and CD8^+^ T lymphocytes

In ACS patients, proportions of CD4^+^ T lymphocytes on days 1, 3 and 7 were significantly decreased compared with IAH patients (25.46 ± 8.36% vs. 37.28 ± 7.06%, 31.26 ± 8.01% vs. 41.76 ± 7.79% and 31.01 ± 9.20% vs. 41.50 ± 9.00%, *P* = 0.000, respectively), while proportions of CD8^+^ T cells showed no significant difference between the two groups on all three days. A significant reduction of the CD4^+^ / CD8^+^ ratio on day 1 was observed in ACS patients compared with IAH ones (1.48 ± 0.51 vs. 1.86 ± 0.49, *P* < 0.01), but not on day 3 and day 7 (Fig 1).

**Fig 2:**
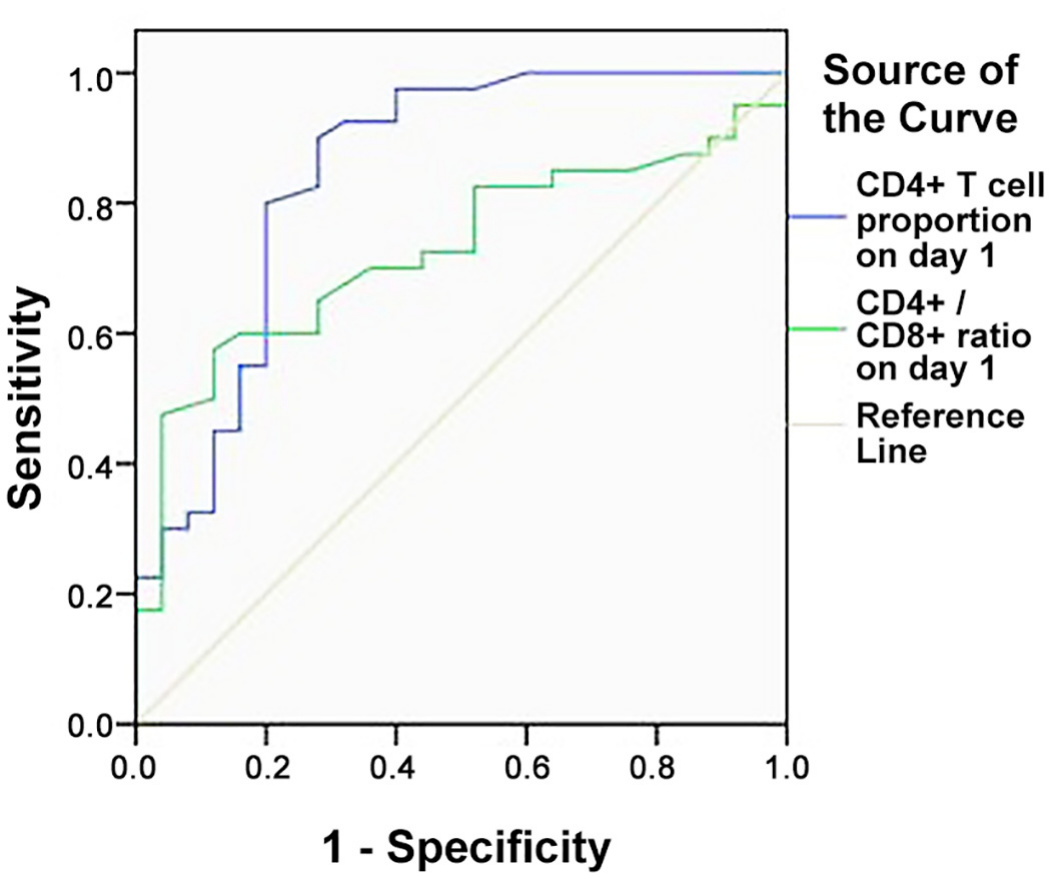
ROC curve of CD4^+^ T cell proportion versus CD4^+^ / CD8^+^ ratio on day 1 in predicting ACS.

To determine early predictors of ACS, we used ROC curves to calculate the optimal cutoff values for the proportion of CD4^+^ T lymphocytes and for the CD4^+^ / CD8^+^ ratio on day 1. It was shown that proportion of CD4^+^ T lymphocyte on day 1 presented an area under the curve (AUC) of 0.774, with a sensitivity of 82.5% and specificity of 72.0%. The optimal threshold was 30.3%. However, the CD4^+^ / CD8^+^ ratio on day 1 showed an AUC of 0.711, with an optimal threshold of 1.48 (Table 2; Fig 2).

**Table 2:** ROC analysis of CD4^+^ T cell proportion, CD4^+^ / CD8^+^ ratio, CTSI scores, and APACHE II scores in diagnosing ACS.

	AUC (95% CI)	Cut—off value	Sensitivity (%)	Specificity (%)
CD4^+^ T cell proportion on day 1	0.774 (0.644–0.903)	< 30.3%	82.5	72.0
CD4^+^ / CD8^+^ ratio on day 1	0.711 (0.582–0.840)	< 1.48	70.0	68.0
APACHE II score on day 1	0.725 (0.610–0.840)	≥ 9.50	72.2	65.0
APACHE II score on day 7	0.696 (0.576–0.815)	≥ 7.50	66.7	65.0
CTSI score on day 1	0.558 (0.427–0.689)	≥ 5.50	30.6	82.5
CTSI score on day 7	0.684 (0.563–0.805)	≥ 4.50	41.7	87.5

AUC: area under the curve; CI: confidence intervals.

### APACHE II score

**Fig 3:**
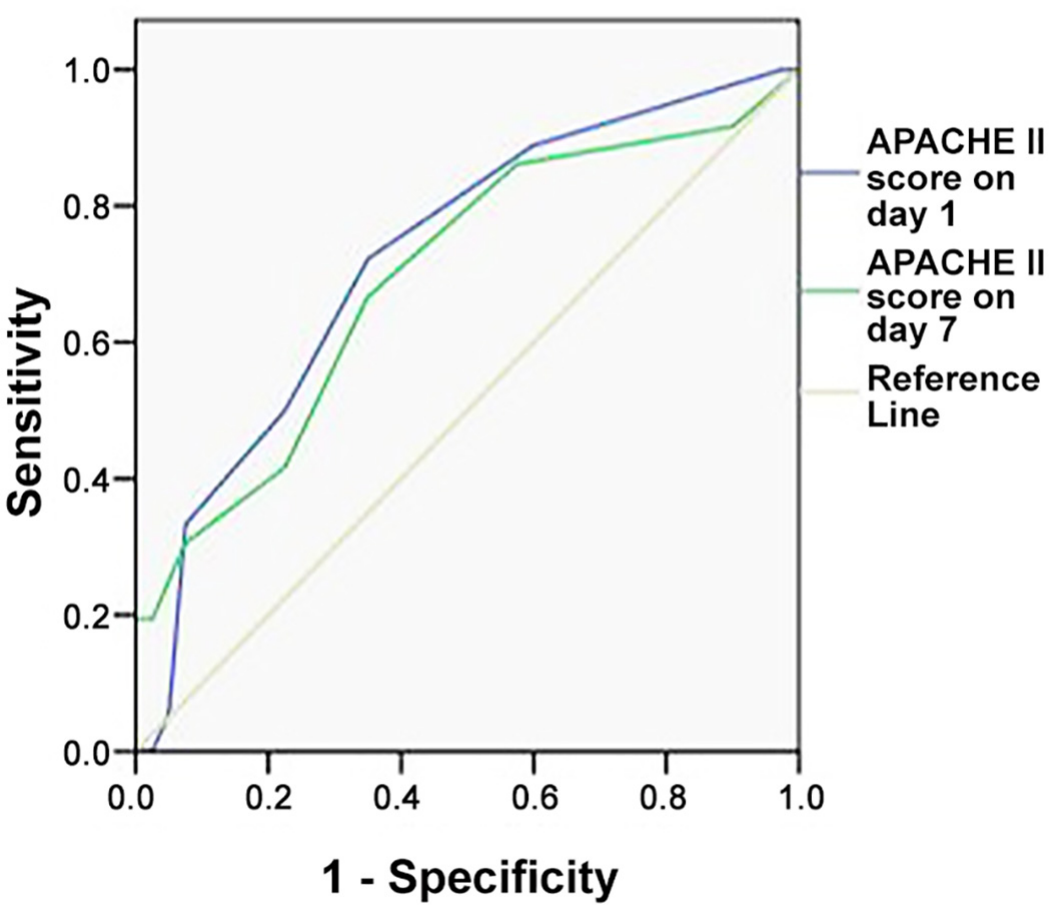
ROC curve of APACHE II score on day 1 versus APACHE II score on day 7 in predicting ACS.

The APACHE II score in ACS patients on day 1 was 10.50 ± 1.48, which was significantly higher than that in IAH patients (9.30 ± 1.56, *P* < 0.01). A significant difference was as well noticed on day 7 (ACS: 9.03 ± 3.01 vs. IAH: 7.15 ± 1.53, *P* < 0.01) (Table 1). ROC analysis was performed for the APACHE II scores on days 1 and 7 (Table 2; Fig 3).

### CTSI score

**Fig 4:**
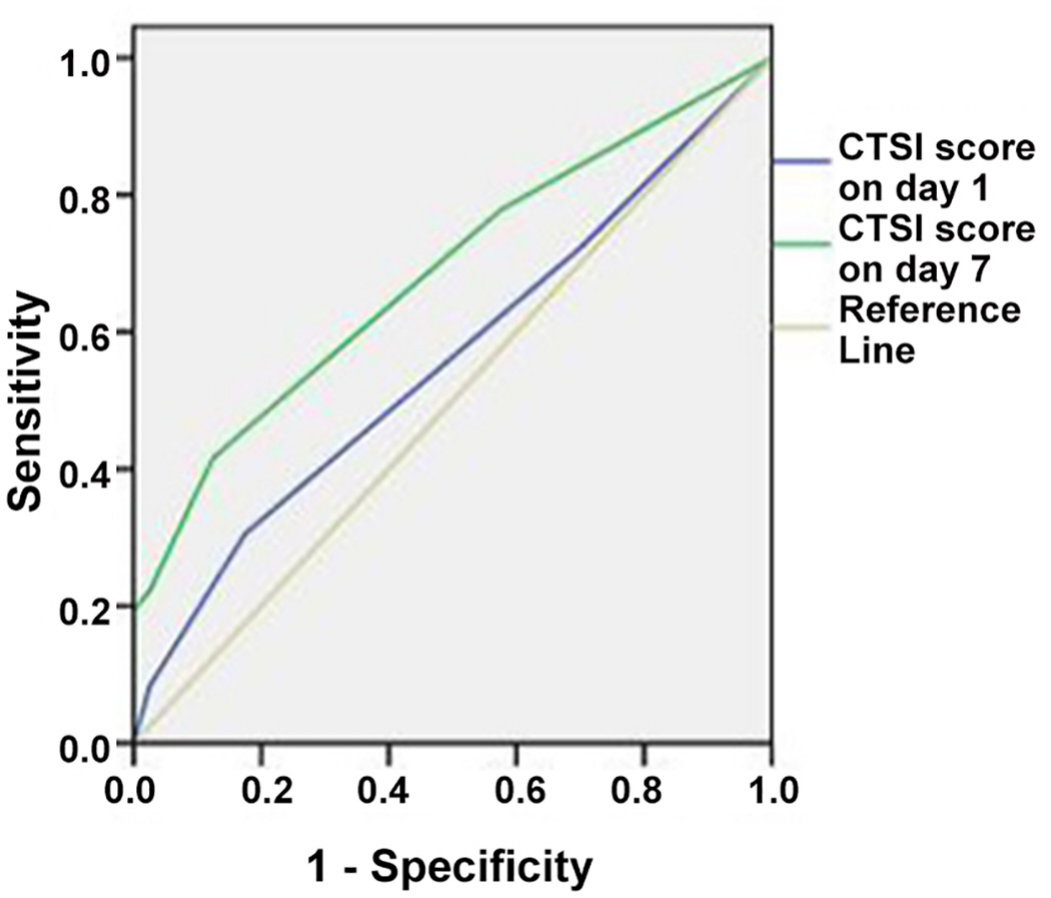
ROC curve of CTSI score on day 1 versus CTSI score on day 7 in predicting ACS.

The CTSI score in IAH patients was significantly decreased from 4.90 ± 0.74 to 3.73 ± 0.75 on day 7 after hospitalization (*P* < 0.01), while that in ACS patients showed no significant difference (5.11 ± 0.92 vs. 4.78 ± 1.71, *P* = 0.306). Besides, the CTSI score in ACS patients on day 7 was significantly higher than that in IAH patients (4.78 ± 1.71 vs. 3.73 ± 0.75, *P* = 0.001) (Table 1). ROC analysis showed that CTSI score on day 7 presented an AUC of 0.684, with a sensitivity of 41.7% and specificity of 87.5% (Table 2; Fig 4).

## Discussion

SAP injures the pancreas itself as well as the surrounding organs. Studies show that 11–30% of SAP patients suffer from abdominal compartment syndrome (ACS), which has a high mortality rate of 30–60%, despite the various treatment protocols [11,22]. It is well accepted that patients suffering from SIRS have a high risk of developing ACS [11–13].

Systemic inflammation is activated by the innate and adaptive immune systems. The innate immune system is directed by monocytes, macrophages and neutrophils, while the adaptive immune system is mainly made up of CD4^+^ T and CD8^+^ T lymphocytes. Pancreatic inflammation triggers the innate immune system, and induces a cytokine cascade. The adaptive immune system is activated by cytokines and antigens. Exaggerated and uncontrollable response leads to SIRS, which can eventually progress into IAH or ACS [11–12, 23].

The decrease of peripheral blood CD4^+^ T cells in the course of AP has been reported previously. Pezzilli *et al*. [24] noticed a significant decrease of both CD4^+^ and CD8^+^ T cells in AP patients, and the severity was related with the number of complications and the clinical outcomes. Curley and colleagues found a significant reduction in the proportion of CD4^+^ T cells in SAP with local complications, such as abscess, necrosis or pseudocyst, compared with the mild form [25]. Besides, CD4^+^ T cells were reported to be further decreased in SAP compared with the mild form, as diagnosed according to serum CRP level, the Ranson’s score, and the Balthazar’s criteria, while CD8^+^ T cells remained normal [26].

ACS is the main cause of early death in SAP patients, so we focused on the relationship between ACS and peripheral T lymphocyte subgroups in this study. We observed that peripheral CD4^+^ cells was significantly decreased in a week-long period after hospitalization in ACS patients compared with IAH ones, indicating that sustained lower levels of CD4^+^ T cells may be related to the progress of ACS and lead to poor outcomes.

Currently, the reasons for the decrease of peripheral CD4^+^ T lymphocytes in SAP remain unclear. One of the possible reasons is an excessive elimination of lymphocytes by apoptosis [26–27]. Another possible explanation is that the activated peripheral CD4^+^ T lymphocytes migrate to the inflammatory organs, such as the pancreas, kidney and lung [15,28], but the precise mechanism needs to be systematically studied. In our study, at a cutoff value of 30.3% of CD4^+^ T cells, ACS was predicted with a sensitivity of 82.5% and a specificity of 72.0%.

Early prediction of ACS contributes to the proper administration of SAP patients, thus decreases the mortality rate. To our knowledge, it is the first time that the peripheral CD4^+^ T lymphocyte proportion and the CD4^+^ / CD8^+^ ratio could be used as early predictive factors for ACS in SAP patients.

CRP is a pro-inflammatory cytokine, and mainly induced by Interleukin-6 (IL-6), which is produced by monocytes and fibroblasts. There is evidence that serum level of CRP correlates with the severity of AP, and predict the critical course of AP combined with IAP [29]. In this study, CRP level was significantly higher on day 7 in the ACS group than the IAH group, indicating that persistent high level of CRP may associate with ACS.

According to the 1992 Atlanta criteria, APACHE II score system may be used to assess the severity during the course of AP, and the severe category was defined as a score ≥ 8 [16]. However, another study [29] found that APACHE II score ≥ 11 is the best predictor of SAP within 24 hours of hospital admission. Gurleyik *et al*. [6] indicated that CTSI score > 3 had better sensitivity and specificity for SAP than either CRP value or APACHE II score. In the present study, the APACHE II scores on days 1 and 7, and CTSI score on day 7 respectively showed a significant different between the ACS and IAH groups, but they showed less abilities to predict the severity of AP than the proportion of CD4^+^ T lymphocytes on day 1. Therefore, the proportion of CD4^+^ T lymphocytes on day 1 is a better predictor for ACS in SAP patients.

There were several limitations in our study. First, in this retrospective study, the number of patients was limited (n = 76), and moderately SAP (MSAP) patients without IAH or ACS, as well as mild AP (MAP) and healthy controls were not included. All patients enrolled are Chinese, race and ethnicity might have important bias on T cell subsets. Second, we lost a part of patients for CD4^+^ / CD8^+^ analyses, and did not assess peripheral blood lymphocyte count and parameters after clinical remission. Furthermore, the patients with any signs of bacterial infections were not excluded, and we did not perform subgroup analyses of the CD4^+^ T cell levels in different etologies. Therefore, a prospective study with large samples including both Chinese and other populations should be performed to investigate the true value of CD4^+^ T lymphocytes in predicting ACS.

## Conclusions

This study indicated that the decrease of CD4^+^ T lymphocytes in the early phase of SAP is related to the progression of ACS. Reduced CD4^+^ T cells may act as a potential predictor of ACS in SAP. The underlying mechanism demands for further clarification in a prospective multicenter study.

## Supporting Information

S1 Table Modified Marshall scoring system for organ dysfunction. (DOCX)

S2 Table Relevant data supporting the findings described in manuscript. (XLSX)
