# Supplementary material for: The Decrease of Peripheral Blood CD4+ T Cells Indicates Abdominal Compartment Syndrome in Severe Acute Pancreatitis
Source: PLoS One. 2015 Aug 19;10(8):e0135768. doi: 10.1371/journal.pone.0135768 (PMC4545887; doi:10.1371/journal.pone.0135768)
Supplement: S1 Table — (DOCX) [file pone.0135768.s001.docx]

**S1 Table Modified Marshall scoring system for organ dysfunction**

| Organ system | Score | | | | |
| --- | --- | --- | --- | --- | --- |
|  | **0** | **1** | **2** | **3** | **4** |
| Respiratory (PaO_2_/FiO_2_) | >400 | 301–400 | 201–300 | 101–200 | ≤101 |
| Renal* | | | | | |
| (serum creatinine, μmol/l) | ≤134 | 134–169 | 170–310 | 311–439 | >439 |
| (serum creatinine, mg/dl) | <1.4 | 1.4–1.8 | 1.9–3.6 | 3.6–4.9 | >4.9 |
| Cardiovascular (systolic blood pressure, mm Hg)† | >90 | <90, fluid responsive | <90, not fluid responsive | <90, pH<7.3 | <90, pH<7.2 |
| For non-ventilated patients, the FiO_2_ can be estimated from below: | | | | | |
| Supplemental oxygen (l/min) | **FiO_2_ (%)** |  | | | |
| Room air | 21 |  | | | |
| 2 | 25 |  | | | |
| 4 | 30 |  | | | |
| 6-8 | 40 |  | | | |
| 9-10 | 50 |  | | | |

A score of 2 or more in any system defines the presence of organ failure.

*A score for patients with pre-existing chronic renal failure depends on the extent of further deterioration of baseline renal function. No formal correction exists for a baseline serum creatinine ≥134 μmol/l or ≥1.4 mg/dl.

†Off inotropic support.
